# Spectrally selective fluorescence imaging of *Chlorobaculum tepidum* reaction centers conjugated to chelator-modified silver nanowires

**DOI:** 10.1007/s11120-017-0455-y

**Published:** 2017-10-31

**Authors:** Dorota Kowalska, Marcin Szalkowski, Khuram Ashraf, Justyna Grzelak, Heiko Lokstein, Joanna Niedziolka-Jonsson, Richard Cogdell, Sebastian Mackowski

**Affiliations:** 1 Institute of Physics, Faculty of Physics, Astronomy and Informatics, Nicolaus Copernicus University, Grudziadzka 5, Torun, Poland; 2 Institute of Molecular, Cell & Systems Biology, Glasgow Biomedical Research Centre, University of Glasgow, 120 University Place, Glasgow, G12 8TA Scotland, UK; 3 Department of Physiology and Cellular Biophysics, Columbia University, Russ Berrie Pavilion, 1150 St. Nicholas Avenue, New York, NY 10025 USA; 4 Department of Chemical Physics and Optics, Charles University, Ke Karlovu 3, Prague, Czech Republic; 5 Institute of Physical Chemistry, Polish Academy of Sciences, Kasprzaka 44/52, Warsaw, Poland; 6 Baltic Institute of Technology, Al. Zwycięstwa 96/98, Gdynia, Poland

**Keywords:** Plasmonic enhancement, Bacteriochlorophyll, Fluorescence, Silver nanowires, Conjugation

## Abstract

A polyhistidine tag (His-tag) present on *Chlorobaculum tepidum* reaction centers (RCs) was used to immobilize photosynthetic complexes on a silver nanowire (AgNW) modified with nickel-chelating nitrilo-triacetic acid (Ni-NTA). The optical properties of conjugated nanostructures were studied using wide-field and confocal fluorescence microscopy. Plasmonic enhancement of RCs conjugated to AgNWs was observed as their fluorescence intensity dependence on the excitation wavelength does not follow the excitation spectrum of RC complexes in solution. The strongest effect of plasmonic interactions on the emission intensity of RCs coincides with the absorption spectrum of AgNWs and is observed for excitation into the carotenoid absorption. From the absence of fluorescence decay shortening, we attribute the emission enhancement to increase of absorption in RC complexes.

## Introduction

Photosynthetic organisms, including bacteria, algae, and plants, can efficiently capture sunlight and convert it into biologically useful forms of chemical energy. From the molecular point of view, these energy conversion reactions take place initially in two types of chlorophyll-binding pigment-protein complexes: light-harvesting antennae, responsible for absorbing photons and transferring energy, and reaction centers (RCs), where the conversion of the energy delivered by the light-harvesting complexes takes place. In particular, RCs are responsible for separating electric charges across the photosynthetic membrane. Nature has optimized this process for high quantum efficiency, where the number of charges separated per absorbed photon is close to unity. This optimization is helped by efficient excitation energy transfer from antenna complexes to RCs, followed by the highly efficient charge separation in the RCs (Blankenship 2002; Pessarakli 2005). There are several types of photosynthetic reaction centers, including bacterial RCs and reaction centers of higher plants, photosystem I (PSI) and photosystem II (PSII) (Nagy et al. 2014). Their unique properties have stimulated intense research focused on employing these photochemically active biomolecules as potential building-blocks for photosensors (Govorov and Carmeli 2007; Terasaki et al. 2007; Terasaki 2016), biosensors for detection of, e.g., herbicides (Ventrella et al. 2010; Swainsbury et al. 2014), gating elements for phototransistors (Terasaki et al. 2007; Frolov et al. 2008), and photovoltaic devices (Mershin et al. 2012; Ocakoglu et al. 2014; Nguyen and Bruce 2014; Friebe et al. 2016). Significant part of this work has been directly connected with the development of novel methods of biochemical modification of proteins, adjusting ways of surface functionalization, and applying various experimental techniques for exploiting the properties of such designed hybrids.

On the other hand, in addition to devising protocols for attaching pigment-protein complexes to surfaces for charge transfer or other biochemical functions, it is also possible to couple them with metallic nanostructures, primarily in order to tune their optical properties (e.g., Mackowski 2010). Namely, it is well known that interactions between metallic nanoparticles and fluorescent molecules can result in strong enhancement of both absorption and fluorescence rates, the latter leading to much shorter lifetimes of excited states and much higher emission intensities. The broad spectrum of research on metallic nanostructures and plasmon resonances associated therewith has been carried out, and it includes nanooptics, sensor design, nanoscale light manipulation, and bioimaging (Anger et al. 2006; Lakowicz 2006; Novotny and Hecht 2006; Bharadwaj et al. 2007). The concept of plasmonic enhancement has been recently extended towards studying interactions between metallic nanostructures and multichromophoric photosynthetic complexes (Brecht et al. 2012; Kowalska et al. 2013; Czechowski et al. 2014; Friebe et al. 2016; Szalkowski et al. 2016; Maćkowski et al. 2016). Depending on the photosynthetic complex and the type of metallic nanostructure used, the actual enhancement factors can vary from only a few-fold to a few-hundred-fold. For algal peridinin-chlorophyll *a*-protein (PCP) deposited on a silver island film (SIF), a six-fold fluorescence intensity enhancement has been observed (Mackowski et al. 2008), whereas for the Fenna-Matthews-Olson (FMO) protein and cyanobacterial photosystem I coupled to similar substrates, the fluorescence can be enhanced by a factor of 40 and 200, respectively (Czechowski et al. 2014; Szalkowski et al. 2016). On the other hand, for photosynthetic complexes deposited on gold nanorods, the enhancement factors may also reach a few hundred (Wientjes et al. 2014), and it has also been shown that for anisotropic plasmonic structures the actual enhancement factors depend on the excitation wavelength (Bujak et al. 2014). While in some cases such large enhancement factors can be attributed predominantly to enhanced emission rates, as indicated by shortening of the excited state lifetimes, it has also been suggested that interaction between metallic nanostructures and photosynthetic complexes can lead to plasmon-induced activation of excitation (and emission) channels absent in isolated photosynthetic complexes (Czechowski et al. 2014; Mackowski et al. 2016). Importantly, in none of these experiments, aimed at studying the effect of plasmonic excitations, proteins were specifically attached to the surfaces, in contrast, they were randomly distributed, regarding both the distance to metallic nanoparticles and their mutual orientation.

Immobilization of biomolecules with defined orientation on both, chemically modified and non-functionalized solid substrates, has been widely applied for studying photocurrent and electronic activities of functional photosynthetic complexes assembled onto metal electrodes (Badura et al. 2006; den Hollander et al. 2011; Kondo et al. 2012; Stieger et al. 2014; Friebe et al. 2016). The RCs were deposited on electrode surfaces with the aim of optimizing the distance from the electrode for efficient electron tunneling between the protein-embedded electron-transfer cofactors and the metal surface. For that purpose, different kinds of RCs were attached to gold electrodes modified with nickel-chelating nitrilo-triacetic acid (NTA) alkanethiol complex using the unique chain of six histidine residues (His-tag) present on the protein (Das et al. 2004; Krassen et al. 2009; Sugiyama et al. 2016).

In this work, we obtained a layer of RCs from the green sulfur bacterium *Chlorobaculum (Cb.) tepidum* on thiolated silver nanowires (AgNWs) via Ni-NTA based conjugation, and demonstrated plasmonic interactions in such a hybrid nanostructure. The results of wide-field fluorescence microscopy demonstrate qualitatively successful binding of His-tagged RCs to the Ni-NTA modified surface of AgNWs. The interactions in this system were probed using spectrally- and temporally-resolved confocal fluorescence microscopy. The emission enhancement of RCs conjugated to the AgNWs with no measurable effect of plasmon excitation on fluorescence lifetimes suggests that plasmonic excitations in silver nanowires modify primarily absorption rates in photosynthetic complexes.

## Materials and methods

*Chlorobaculum tepidum* cultures were grown, and RCs were isolated and purified, as described previously (Azai et al. 2011; Ashraf 2014). The buffer used for the RCs contained 20 mM TRIS, pH 8.0, 150 mM NaCl, and 0.05% β-dodecyl maltoside (Glycon, Germany). The RCs were equipped with a His-tag. Silver nanowires were synthesized using a polyol process in the presence of ethylene glycol, copper seeds, and poly(vinyl pyrrolidone) polymer (PVP) (Sun et al. 2002). The morphology of AgNWs was examined using scanning electron microscopy (Kowalska et al. 2013), the optical properties of metallic nanoparticles, and RCs in solutions were studied using absorption and steady-state fluorescence spectroscopy.

In order to facilitate efficient attachment of His-tagged RCs, the surface of AgNWs was modified by nickel-chelating nitrilo-triacetic acid (Ni-NTA) as described previously (Kondo et al. 2012). After functionalization, AgNWs were incubated overnight at room temperature with RCs in buffer solution, pH 8.0. Next, the solution of immobilized RCs on AgNWs was purified by carefully rinsing and centrifuging three times in buffer solution. In this way, we supposedly remove any unattached protein from the solution.

Absorption spectra were measured using a Varian Cary 50 UV–Vis spectrophotometer. Fluorescence spectra of RC solutions were recorded using a Fluorolog-3 spectrofluorometer (JobinYvon) equipped with a Xenon lamp for excitation and a double grating monochromator. The signal was detected using a thermoelectrically cooled photomultiplier tube with a dark current less than 100 cps.

Fluorescence intensity maps were acquired using a Nikon Eclipse Ti-S inverted wide-field microscope equipped with Andor iXon Du-888 EMCCD detector cooled to − 80 °C for low dark current counts. For the excitation, a sequence of LED illuminators was used with wavelengths of 365, 407, 475, 533, and 630 nm. The excitation light was reflected by a dichroic mirror (Semrock, FF775-Di01) to the microscope objective lens (Plan Apo, 100×, NA = 1.4, oil immersion, Nikon). The excitation power after the dichroic mirror was 200 µW for each of the excitation wavelengths. The RC emission was filtered using a 750 nm longpass filter (Thorlabs, FELH0750) and a narrowband pass filter (Thorlabs, FB850/40). Acquisition time was 1 s, electron multiplying gain was 50, and the size of fluorescence images was approximately 50 × 50 µm. Around 50 sets of fluorescence intensity maps of RCs conjugated with AgNWs were collected. Each set contains fluorescence intensity maps for the same area on the sample measured for five different excitation wavelengths, with the sequence starting from 630 nm towards 365 nm. This allows to compare the fluorescence intensity of RCs@AgNW for the particular AgNW as a function of the excitation wavelength, and thus demonstrate emergence of plasmonic coupling in the hybrid nanostructure. Moreover, the sequence when the sample is first excited with longer wavelengths minimizes any photo-bleaching of the photosynthetic protein.

Spectrally- and time-resolved fluorescence measurements were performed using a home-built confocal fluorescence microscope. For excitation of emission, two lasers with wavelengths of 485 and 640 nm were used. They operate in a pulsed mode with a repetition rate of 20 MHz and average power of 48 μW and 22 μW, respectively. The 640-nm laser was additionally filtered with a shortpass filter (Thorlabs, FES0650) and a bandpass filter (Thorlabs, FB640/10). Samples, containing RCs conjugated with AgNWs were placed on a XYZ piezoelectric stage and the excitation beam was focused onto the surface using an oil-immersion objective 60×, NA = 1.49 (Nikon). In this way, it was possible to place the laser spot with a defined location on the sample. Fluorescence intensity maps were collected with a single photon counting module (PerkinElmer, SPCM-AQRH-14) with the emission filtered by a longpass filter (Thorlabs, FELH0750). The spectra were detected with an Andor iDus 420A-BV CCD detector coupled to an Amici prism applied as a dispersive element. Time-correlated single photon counting technique was used to measure fluorescence decay curves. The setup comprises an SPC-150 module (Becker&Hickl) with a fast avalanche photodiode (idQuantique, id100-50) for detection, and is characterized by a temporal resolution of approximately 100 ps. For time-resolved fluorescence measurements, the RC emission was filtered by a combination of a longpass (Thorlabs, FELH0750) and a bandpass (Thorlabs, FB850/40) filters.

## Results and discussion

**Fig. 1:**
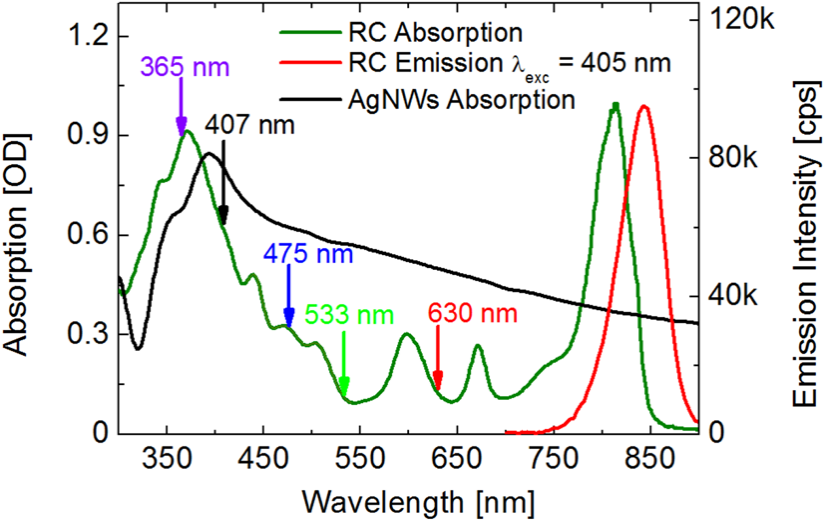
Absorption spectra of RCs (olive), AgNWs (black), and emission spectrum of RCs in buffer solution upon excitation at 405 nm (red curve)

Absorption and emission spectra of RCs in buffer solution are presented in Fig. 1. Carotenoids, i.e., chlorobactene, γ-carotene, and their hydroxylated glucoside esters, absorb light in the region between 430 and 540 nm. Contribution of bacteriochlorophylls *a* (BChl a) to the absorption spectrum of the RCs is shown in two spectral regions from 300 to 430 nm, and between 540 and 850 nm (the Q_x_ and Q_y_ bands are seen around 600 and 810 nm, respectively). A chlorophyll *a* derivative in RC has its Q_y_ band at around 670 nm (Takaichi and Oh-oka 1999). The FMO protein attached to the RCs also contains BChl *a*, and absorbs light in the spectral region from 550 to 645 nm (Olson 2004). Light absorbed by FMO is predominantly transferred to the RCs, although it cannot be excluded that the FMO complexes may also contribute to the emission of the RC complex. The emission spectrum of RCs, presented in Fig. 1, exhibits a broad peak with the maximum at around 840 nm, with a slight shoulder towards shorter wavelengths, which indeed might be due to the FMO emission.

The absorption spectrum of AgNWs, shown in Fig. 1, exhibits a maximum at around 390 nm, corresponding to the plasmon resonance. As discussed previously, the absorption spectrum of the AgNWs is very broad and overlaps very well with both, absorption and emission spectrum of the RCs, thus promoting efficient interactions between the RCs and the plasmon resonances in the metallic nanowires. The arrows in Fig. 1 mark the excitation wavelengths of 365, 407, 475, 533, and 630 nm, used for imaging fluorescence of RC@AgNWs conjugates. The broad range of excitation wavelengths allows to probe the optical properties of the hybrid nanostructure upon exciting various components of RCs, such as carotenoids, RC-BChls *a* and FMO, and probe their separate interactions with metallic nanoparticles.

**Fig. 2:**
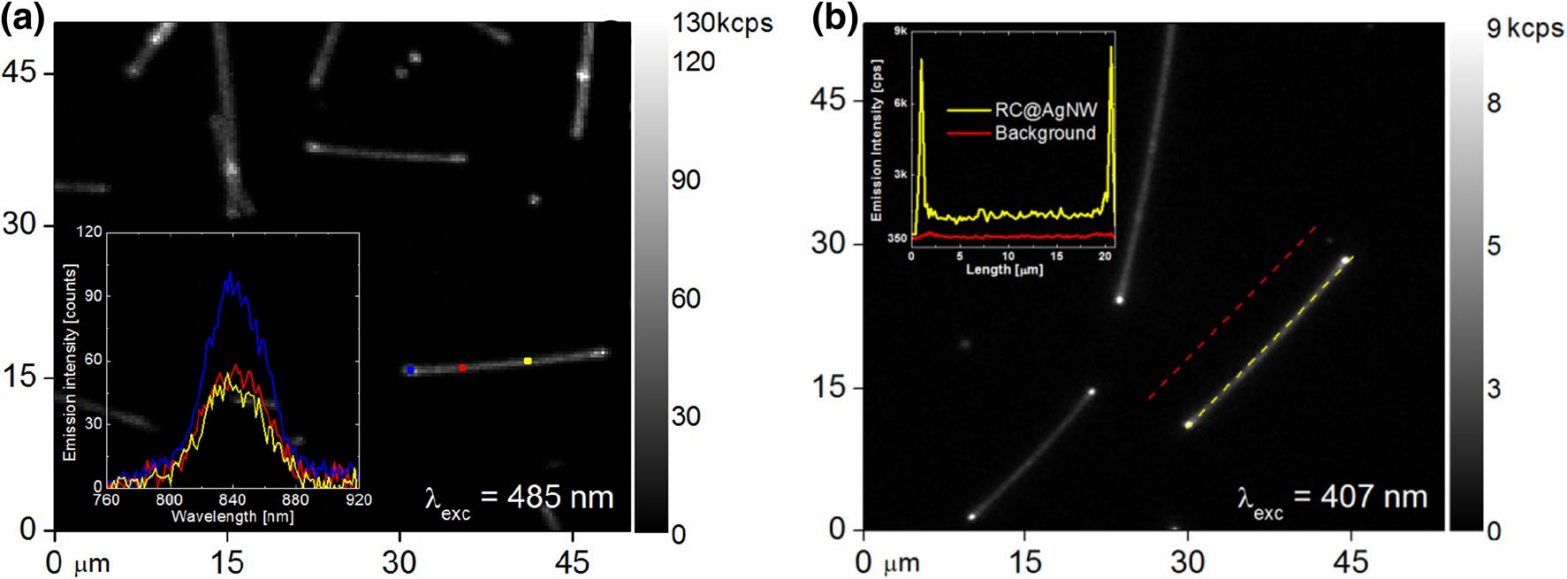
Fluorescence intensity map obtained for RCs conjugated with AgNWs: (**a**) using confocal microscopy, excited at 485 nm. The inset displays emission spectra of RCs conjugated with AgNWs for the selected spots along the nanowire; **b** using wide-field microscopy, excited at 407 nm. The inset presents emission intensity distribution along a single AgNW (yellow) and in the vicinity of a selected AgNW (red)

The optical properties of the conjugate composed of RCs attached to AgNWs were studied using confocal and wide-field fluorescence microscopy. Examples of typical fluorescence images measured using 485 and 407 nm are shown in Fig. 2a, b, respectively. Fluorescence intensity map obtained for the RCs@AgNW conjugate using 485 nm excitation (Fig. 2a) features bright elongated shapes on an essentially dark background. The positions of these elongated shapes correlate perfectly with the positions of the nanowires determined using transmission microscopy of the same sample area (not shown). We ascribe these elongated emitting shapes to the fluorescence of RCs conjugated to AgNWs. Importantly, there is essentially no signal that would originate from areas off the nanowires, indicating that no free protein is left in the solution. We also note that the nanowires themselves which are not covered with fluorescence emitting proteins, exhibit no emission at all. The fluorescence image measured using 407 nm excitation wavelength (Fig. 2b) is qualitatively similar: silver nanowires covered with RC complexes emit light along the nanowires with brighter spots at their ends. The inset in Fig. 2a displays emission spectra of RCs conjugated with AgNWs measured for selected marked spots along the nanowire using confocal fluorescence microscopy. The shape and positions of the fluorescence maxima correspond to the emission spectrum of the RCs in buffer solution, indicating maintained functionality of the protein upon conjugation with metallic nanowires. In agreement with the results of wide-field microscopy, no emission was measured when laser beam was located off the nanowire. The inset in Fig. 2b, where a wide-field fluorescence microscopy image is displayed, shows the emission intensity distribution along a single AgNW (yellow) and in the vicinity of the selected AgNW (red). It can be seen, that the fluorescence intensity of RCs on the nanowire is higher than that of the background, and—with the exception of the ends of the nanowire—is rather uniform. In addition, the emission at the end of AgNWs is substantially stronger that along the nanowire. This effect has been previously observed for instance for light-harvesting complexes (Kowalska et al. 2013) and presumably is attributed to scattering of plasmon excitation at the ends of the nanowires. The results of fluorescence microscopy demonstrate three effects: (1) RCs are specifically attached to AgNWs, (2) the coverage is rather uniform since no dramatic variation in emission intensity along the nanowires is observed, and (3) the emission of RCs conjugated to AgNWs is not quenched, pointing towards the conclusion that the distance between the nanowires and RCs is large enough to inhibit non-radiative energy transfer.

The key question regarding the results displayed in Fig. 2 concerns the emergence of plasmonic interactions in such a conjugate. While the observation of RC emission implies that the complexes are indeed attached and their emission is not quenched, proving the effect of plasmonic excitations on the RCs optical properties of is not trivial for such structures. Indeed, it is difficult to suggest possible reference that would be suitable for comparison, in a similar way as it has been done previously for layered plasmonic hybrid nanostructures (Czechowski et al. 2014; Szalkowski et al. 2016; Maćkowski et al. 2016). To this end, we decided to use our experimental ability to excite the fluorescence with several excitation wavelengths. In fact, we carried out systematic studies of the fluorescence properties of RC@AgNW conjugates using imaging with five different excitation wavelengths, spanning from 365 to 630 nm. These excitation wavelengths cover almost the whole absorption spectrum of the RCs and allow excitation of each particular pigments embedded in the RC protein scaffold. The underlying assumption behind this experiment is that the spectral dependence of fluorescence intensity determined for RC@NWs should be different to the analogous relation measured for RCs in buffer solution. In other words, we expect that plasmonic interactions should affect the spectral dependence of the fluorescence intensities.

**Fig. 3:**
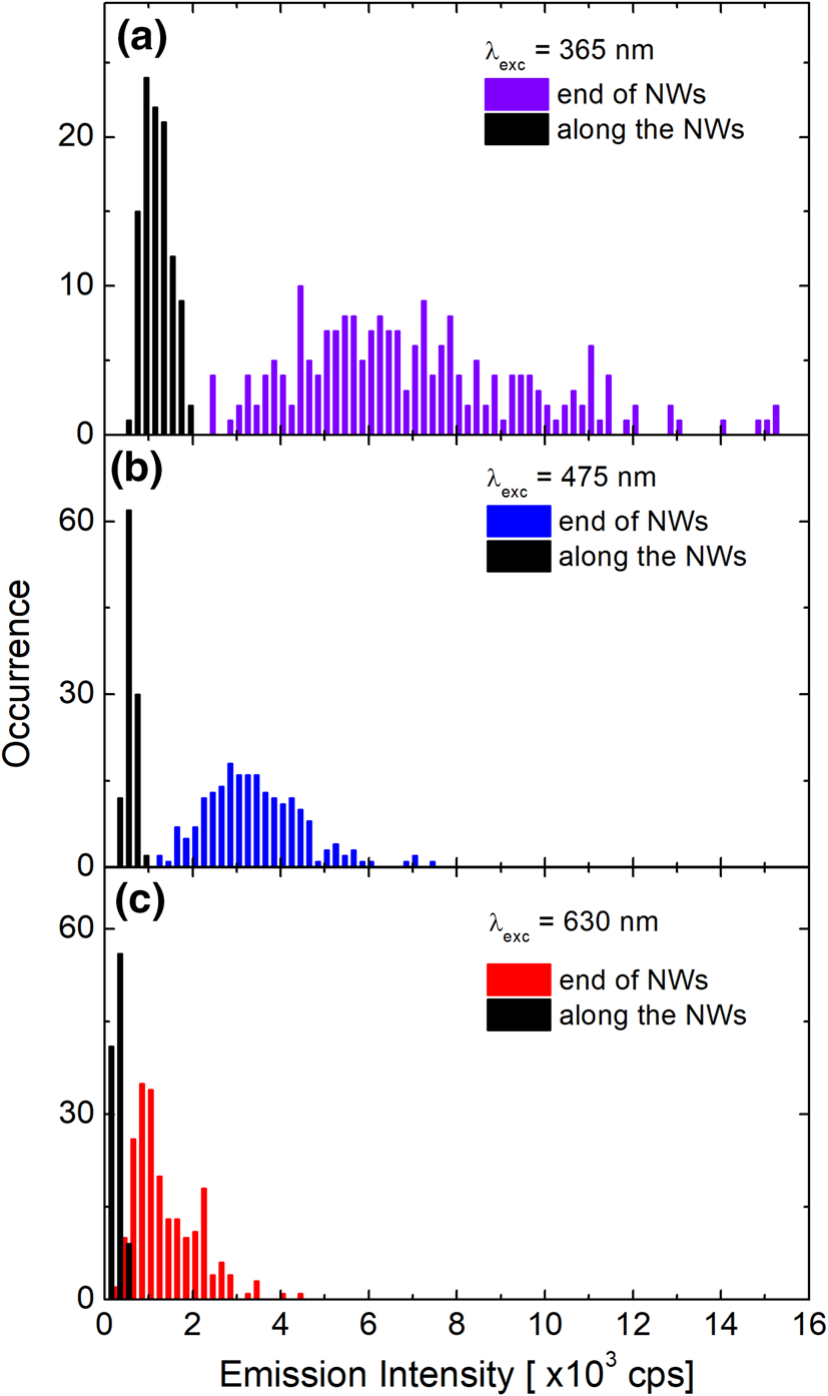
Histograms of fluorescence intensity obtained for RCs conjugated with AgNWs along the NWs (black bars) and at the ends of NWs (violet, cyan and red bars), excited at: **a** 365 nm, **b** 475 nm, and **c** 630 nm

As mentioned already, we collected approximately 50 maps of silver nanowires conjugated with RCs for each excitation wavelength. Thus, we analyzed over a hundred of individual silver nanowires, determining the average intensity of RC emission along the nanowire and at its ends. In Fig. 3, we compare the histograms of fluorescence intensity obtained for RCs conjugated with AgNWs for three excitation wavelengths. The emission intensities of the RC@AgNWs measured along the nanowires are displayed as black bars and those obtained at the ends of NWs as violet, cyan, and red bars for excitations at (a) 365 nm, (b) 475 nm, and (c) 630 nm, respectively. The average values of fluorescence intensity of RC@AgNWs along the nanowires are approximately 1100, 540, and 250 cps for excitations at 365, 475, and 630 nm, respectively. Corresponding intensities determined for the nanowire ends are equal 7100, 3500, and 1400 cps. The experimental data show that the fluorescence intensity of RCs is significantly higher at the ends of the nanowires as compared to the intensity measured along the nanowires. Since the nanowires feature sharp crystalline ends, they might be considered as a source of an additional electromagnetic field enhancement due to the antenna effect. On the other hand, the strong plasmonic effect observed at the ends of the nanowires can also be due to enhanced scattering of plasmons (Olejnik et al. 2013).

**Fig. 4:**
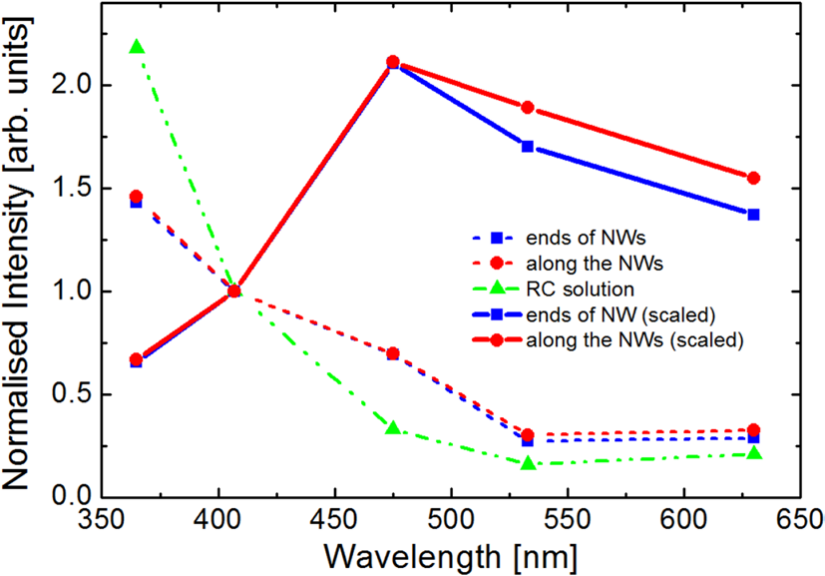
Comparison of the fluorescence intensity of an RC solution (green triangles) and averaged emission intensity of RC@AgNWs at the ends of NWs (blue squares) as well as along the NWs (red circles). Solid lines correspond to emission intensity of RC@AgNWs scaled to the excitation spectrum of the RC solution

Spectrally-resolved fluorescence imaging allows for demonstration of plasmonic effects in the RCs@AgNWs conjugate, as shown in Fig. 4. Fluorescence intensity of RC emission in solution for each varied excitation wavelengths is shown as green triangles. These values were obtained directly from the fluorescence excitation spectrum, which was detected while keeping constant excitation power for each wavelength. On the other hand, the intensities for RCs@AgNWs conjugates were measured using wide-field microscopy for identical excitation powers of the five LED illuminators used. The resulting values obtained along the NWs and at their ends are shown in Fig. 4 as red and blue symbols connected with dashed lines, respectively. Further, in order to facilitate comparison, we normalized the intensities to the values calculated for the excitation at 407 nm. Clearly, the resulting dependence is different to that of the RCs in solution, what is better visible after normalization of the intensities at 407 nm, as plotted in Fig. 4. While the fluorescence intensity of RCs in solution is higher when excited in the UV range (365 nm excitation), excitation at 485 nm results in a reversed relation: the emission is approximately twofold enhanced as compared to RCs in solution. By adjusting the values obtained for RCs@AgNWs by dividing them by the excitation efficiency, we can separate the fluorescence intensity into a native component (identical to that of RCs in solution) and the contribution associated with plasmonic excitations. The result is displayed as symbols connected by solid lines in Fig. 4. The scaling of emission intensities of RCs@AgNWs to the intensities of RCs in solution emphasizes the strong effect of plasmon excitations in AgNWs on the emission of the RCs in the conjugated structure. Namely, when conjugated to AgNWs, the fluorescence intensity of RCs exhibits a non-monotonic behavior with the maximum intensity at around 470 nm, which corresponds roughly to the maximum of plasmon resonance of silver nanowires, which is expected to be red-shifted upon coating with proteins. We can therefore conclude that not only we were able to specifically conjugate RCs to silver nanowires, but also that in such a hybrid nanostructure the plasmonic coupling emerges between specific cofactors embedded in the protein and the silver nanowires.

Fluorescence enhancement of RC complexes has been previously studied in structures containing the RCs deposited in a polymer layer on silver island film (SIF) (Mackowski et al. 2016) and rather large values of enhancement factors were demonstrated. The spectral dependence of the enhancement factors is quite similar to the relation observed in the current study, although the morphology of the conjugated structure is qualitatively different. Perhaps this is due to the fact, that the shape of absorption spectrum of the SIF is similar to that of silver nanowires. Although for RCs conjugated with silver nanowires, it is rather difficult to determine the actual values of enhancement factors, the similarities between the two plasmonic nanostructures might—to some degree—suggest that the order of magnitude should also be comparable.

**Fig. 5:**
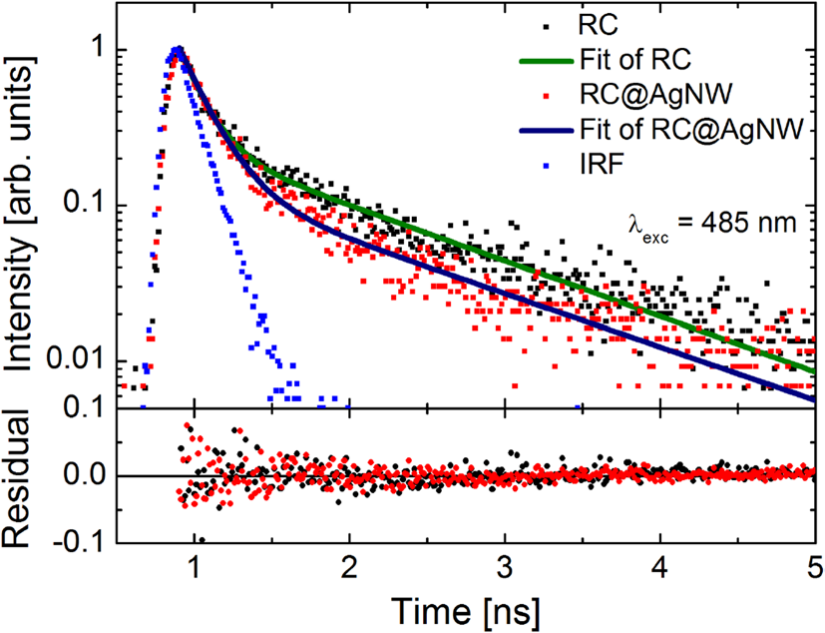
Fluorescence decay curves measured for RCs in solution (black dots) and RCs conjugated with AgNWs (red dots) upon excitation at 485 nm. The instrument response function (IRF) is shown in blue

Important information about the mechanism responsible for plasmonically induced enhancement in the conjugated RCs@AgNWs hybrid nanostructure can be deduced from time-resolved fluorescence spectroscopy (Ray et al. 2006; Mackowski et al. 2008). Normalized fluorescence transients obtained for RCs in solution (black dots) and RCs@AgNWs (red dots) for 485 nm excitation are compared in Fig. 5. Despite differences in the relative intensities, the overall shapes of the decay curves measured for RCs and RCs@AgNWs are essentially similar. For both excitation wavelengths of 485 and 640 nm, we obtained decay times of 150 ps (comparable to our temporal resolution) and 1.3 ns for the shorter and longer components of the fitted bi-exponential curves (olive line for pure RCs and dark blue line for RC@AgNWs), respectively. While we do not observe any measurable shortening of the decays of the fluorescence of RCs conjugated to AgNWs, this could mean that the strong effect of the excitation wavelength on the fluorescence intensity, as determined from the fluorescence intensity maps, is not related to significant changes in the fluorescence dynamics of excited states. Rather, the results of time-resolved fluorescence measurements suggest that the increase of emission intensity is primarily associated with enhancement of RC absorption upon coupling to silver nanowires.

## Conclusions

In conclusion, RCs from *Cb. tepidum* were successfully immobilized on chelator-modified AgNWs using polyhistidine tags. In this hybrid nanostructure, we demonstrate enhancement of fluorescence by analyzing its spectral dependence. The strongest enhancement of emission intensity is measured for excitation at 475 nm, which is close to the maximum of plasmon resonance in AgNWs and corresponds to absorption of carotenoids. The fluorescence transients obtained for RCs immobilized to AgNWs are similar to that of RCs in solution, thus we assign the observed fluorescence enhancement to increase of absorption in the photosynthetic complex, related perhaps to more efficient excitation energy transfer from carotenoids to emitting bacteriochlorophylls.

## References

[CR1] Anger P, Bharadwaj P, Novotny L (2006). Enhancement and quenching of single-molecule fluorescence. Phys Rev Lett.

[CR2] Ashraf KU (2014) Studies of the green sulphur bacterial reaction centre from *Chlorobaculum tepidum*. PhD thesis, University of Glasgow

[CR3] Azai C, Kim K, Kondo T (2011). A heterogeneous tag-attachment to the homodimeric type 1 photosynthetic reaction center core protein in the green sulfur bacterium *Chlorobaculum tepidum*. Biochimica et Biophysica Acta (BBA) - Bioenergetics.

[CR4] Badura A, Esper B, Ataka K (2006). Light-driven water splitting for (Bio-)hydrogen production: photosystem 2 as the central part of a bioelectrochemical device. Photochem Photobiol.

[CR5] Bharadwaj P, Anger P, Novotny L (2007). Nanoplasmonic enhancement of single-molecule fluorescence. Nanotechnology.

[CR6] Blankenship RE (2002) Molecular mechanisms of photosynthesis, 1st edn. Blackwell Science

[CR7] Brecht M, Hussels M, Nieder JB (2012). Plasmonic interactions of photosystem I with Fischer patterns made of Gold and Silver. Chem Phys.

[CR8] Bujak L, Czechowski N, Piatkowski D (2011). Fluorescence enhancement of light-harvesting complex 2 from purple bacteria coupled to spherical gold nanoparticles. Appl Phys Lett.

[CR9] Bujak Ł, Olejnik M, Brotosudarmo THP (2014). Polarization control of metal-enhanced fluorescence in hybrid assemblies of photosynthetic complexes and gold nanorods. Phys Chem Chem Phys.

[CR10] Czechowski N, Lokstein H, Kowalska D (2014). Large plasmonic fluorescence enhancement of cyanobacterial photosystem I coupled to silver island films. Appl Phys Lett.

[CR11] Das R, Kiley PJ, Segal M (2004). Integration of photosynthetic protein molecular complexes in solid-state electronic devices. Nano Lett.

[CR12] den Hollander M-J, Magis JG, Fuchsenberger P (2011). Enhanced photocurrent generation by photosynthetic bacterial reaction centers through molecular relays, light-harvesting complexes, and direct protein–gold interactions. Langmuir.

[CR13] Friebe VM, Delgado JD, Swainsbury DJK (2016). Plasmon-enhanced photocurrent of photosynthetic pigment proteins on nanoporous silver. Adv Funct Mater.

[CR14] Frolov L, Rosenwaks Y, Richter S (2008). Photoelectric junctions between GaAs and photosynthetic reaction center protein. J Phys Chem C.

[CR15] Govorov AO, Carmeli I (2007). Hybrid structures composed of photosynthetic system and metal nanoparticles: plasmon enhancement effect. Nano Lett.

[CR16] Kondo M, Iida K, Dewa T (2012). Photocurrent and electronic activities of oriented-His-tagged photosynthetic light-harvesting/reaction center core complexes assembled onto a gold electrode. Biomacromolecules.

[CR17] Kowalska D, Krajnik B, Olejnik M (2013). Metal-enhanced fluorescence of chlorophylls in light-harvesting complexes coupled to silver nanowires. Sci World J.

[CR18] Krassen H, Schwarze A, Friedrich B (2009). Photosynthetic hydrogen production by a hybrid complex of photosystem I and [NiFe]-hydrogenase. ACS Nano.

[CR19] Lakowicz JR (2006) Principles of fluorescence spectroscopy, 3rd edn. Springer

[CR20] Mackowski S (2010). Hybrid nanostructures for efficient light harvesting. J Phys-Condes Matter.

[CR21] Mackowski S, Wörmke S, Maier AJ (2008). Metal-enhanced fluorescence of chlorophylls in single light-harvesting complexes. Nano Lett.

[CR22] Maćkowski S, Czechowski N, Ashraf KU (2016). Origin of bimodal fluorescence enhancement factors of *Chlorobaculum tepidum* reaction centers on silver island films. FEBS Lett.

[CR23] Mershin A, Matsumoto K, Kaiser L (2012). Self-assembled photosystem-I biophotovoltaics on nanostructured TiO2 and ZnO. Sci Rep.

[CR24] Nagy L, Magyar M, Szabó T (2014). Photosynthetic machineries in nano-systems. Curr Protein Pept Sci.

[CR25] Nguyen K, Bruce BD (2014). Growing green electricity: progress and strategies for use of photosystem I for sustainable photovoltaic energy conversion. Biochimica et Biophysica Acta (BBA) - Bioenergetics.

[CR26] Novotny L, Hecht B (2006) Principles of nano-optics. Cambridge University Press

[CR27] Ocakoglu K, Krupnik T, Van DB (2014). Photosystem I-based biophotovoltaics on nanostructured hematite. Adv Funct Mater.

[CR28] Olejnik M, Krajnik B, Kowalska D (2013). Imaging of fluorescence enhancement in photosynthetic complexes coupled to silver nanowires. Appl Phys Lett.

[CR29] Olson JM (2004). The FMO protein. Photosyn Res.

[CR30] Pessarakli M (ed) (2005) Handbook of photosynthesis, 2nd edn. CRC Press

[CR31] Ray K, Badugu R, Lakowicz JR (2006). Metal-enhanced fluorescence from CdTe nanocrystals: a single-molecule fluorescence study. J Am Chem Soc.

[CR32] Stieger KR, Feifel SC, Lokstein H, Lisdat F (2014). Advanced unidirectional photocurrent generation via cytochrome c as reaction partner for directed assembly of photosystem I. Phys Chem Chem Phys.

[CR33] Sugiyama M, Fujii K, Nakamura S (2016) Solar to chemical energy conversion: theory and application. Springer International Publishing

[CR34] Sun Y, Yin Y, Mayers BT (2002). Uniform silver nanowires synthesis by reducing AgNO3 with ethylene glycol in the presence of seeds and Poly(Vinyl Pyrrolidone). Chem Mater.

[CR35] Swainsbury DJK, Friebe VM, Frese RN, Jones MR (2014). Evaluation of a biohybrid photoelectrochemical cell employing the purple bacterial reaction centre as a biosensor for herbicides. Biosens Bioelectron.

[CR36] Szalkowski M, Ashraf KU, Lokstein H (2016). Silver island film substrates for ultrasensitive fluorescence detection of (bio)molecules. Photosyn Res.

[CR37] Takaichi S, Oh-oka H (1999). Pigment composition in the reaction center complex from the thermophilic green sulfur bacterium, *Chlorobium tepidum*: carotenoid glucoside esters, menaquinone and chlorophylls. Plant Cell Physiol.

[CR38] Terasaki N (2016). PS-I and PS-II on electrodes for energy generation and photo-sensor. Lect Notes Energy.

[CR39] Terasaki N, Yamamoto N, Tamada K (2007). Bio-photosensor: cyanobacterial photosystem I coupled with transistor via molecular wire. Biochimica et Biophysica Acta - Bioenergetics.

[CR40] Ventrella A, Catucci L, Agostiano A (2010). Herbicides affect fluorescence and electron transfer activity of spinach chloroplasts, thylakoid membranes and isolated Photosystem II. Bioelectrochemistry.

[CR41] Wientjes E, Renger J, Curto AG (2014). Strong antenna-enhanced fluorescence of a single light-harvesting complex shows photon antibunching. Nat Commun.

